# Use of a Participatory Method for Community-Based Brucellosis Control Design in Agro-Pastoral Areas in Tanzania

**DOI:** 10.3389/fvets.2022.767198

**Published:** 2022-02-09

**Authors:** Shingo Asakura, George Makingi, Kunda John, Rudovick Kazwala, Kohei Makita

**Affiliations:** ^1^Veterinary Epidemiology Unit, Graduate School of Veterinary Medicine, Rakuno Gakuen University, Ebetsu, Japan; ^2^Department of Veterinary Medicine and Public Health, College of Veterinary Medicine and Biomedical Sciences, Sokoine University of Agriculture, Morogoro, Tanzania; ^3^One Health Coordination Desk, Prime Minister's Office, Dar es Salaam, Tanzania

**Keywords:** agro-pastoralist, brucellosis, disease control, participatory epidemiology, Tanzania

## Abstract

Brucellosis is widespread in both humans and livestock in many developing countries. The authors have performed a series of epidemiological studies on brucellosis in agro-pastoral areas in Tanzania since 2015, with the aim of the disease control. Previously, the potential of a community-based brucellosis control initiative, which mainly consisted of the sale of cattle with experience of abortion and vaccinating calves, was assessed as being effective and acceptable based on a quantitative approach. This study was conducted to investigate the feasibility of community-based brucellosis control program using participatory rural appraisals (PRAs) and key-informant interviews. Four PRAs were performed together with livestock farmers and livestock and medical officers in 2017. In the PRAs, qualitative information related to risky behaviors for human infection, human brucellosis symptoms, willingness to sell cattle with experience of abortion, and willingness to pay for calf vaccination were collected, and a holistic approach for a community-based disease control project was planned. All of the communities were willing to implement disease control measures. To avoid human infection, education, especially for children, was proposed to change risky behaviors. The findings of this study showed that community-based disease control measures are promising.

## Introduction

Brucellosis is a zoonotic disease of veterinary, public health and economic importance, especially in developing countries (1). In livestock, brucellosis results in reduced productivity through abortion, infertility and low milk production (2). Human brucellosis causes flu-like symptoms, including persistent and irregular fever, malaise, arthralgia and other constitutional symptoms, and results in high-cost treatment and loss of income due to loss of working time (3). In cattle, the disease can be transmitted through aborted fetus, placenta, milk and semen from infected animals (2). For human infection, consumption of unheated meat and dairy products and contact with infected animals are the main transmission routes (4).

Generally, zoonosis control can be achieved effectively by tackling animal reservoirs. Bovine brucellosis control activities consist of surveillance, control of movement, stamping out and vaccination. However, the implementation of these control measures has been poor in sub-Saharan countries (5). In Tanzania, where brucellosis is widespread in both animals and livestock keepers, the control of brucellosis by the national and/or local governments is unfeasible due to limited resources (6, 7). Since 2015, the authors have performed epidemiological research on brucellosis in cattle and humans in agro-pastoral areas in Morogoro region, Tanzania. Those quantitative studies revealed the endemic status of brucellosis in the cattle of the region, with the individual and herd level prevalences 7.0 and 44.4%, respectively (8, 9). Risk factor analysis revealed a strong association between abortion and brucellosis in cattle. In addition, a high willingness to pay 3,000 Tanzanian Shillings (~1.3 USD) for calf *Brucella* vaccinations (89.6%) was observed among cattle farmers, indicating that community-based bovine brucellosis control is potentially feasible (9).

A qualitative research approach, referred to as participatory epidemiology (PE), has become an increasingly important area in epidemiology (10). The use of participatory rural appraisals (PRA) is one of the techniques used in PE and is widely used to collect and evaluate the opinions of a target group (11–13). The participatory approach overcomes the limitations of conventional epidemiological methods, such as high cost, complexity in logistics, and misinterpretation of quantitative information due to the researchers' lack of understanding of the local context (14, 15). Moreover, PRA is an effective method for not only collecting information, but also for ensuring stakeholders' participation in decision making (16). In the veterinary field, participatory methods have been widely used in community-based livestock projects in Africa and Asia since the 1980's (17). Since then, participatory approaches have been refined and subsequently integrated as a sub-discipline in the emerging field of veterinary epidemiology (10, 18).

The objective of the current study was to assess the potential of community-based disease control using PRA as a means of complimenting quantitative information obtained from conventional epidemiological studies.

## Materials and Methods

**Figure 1:**
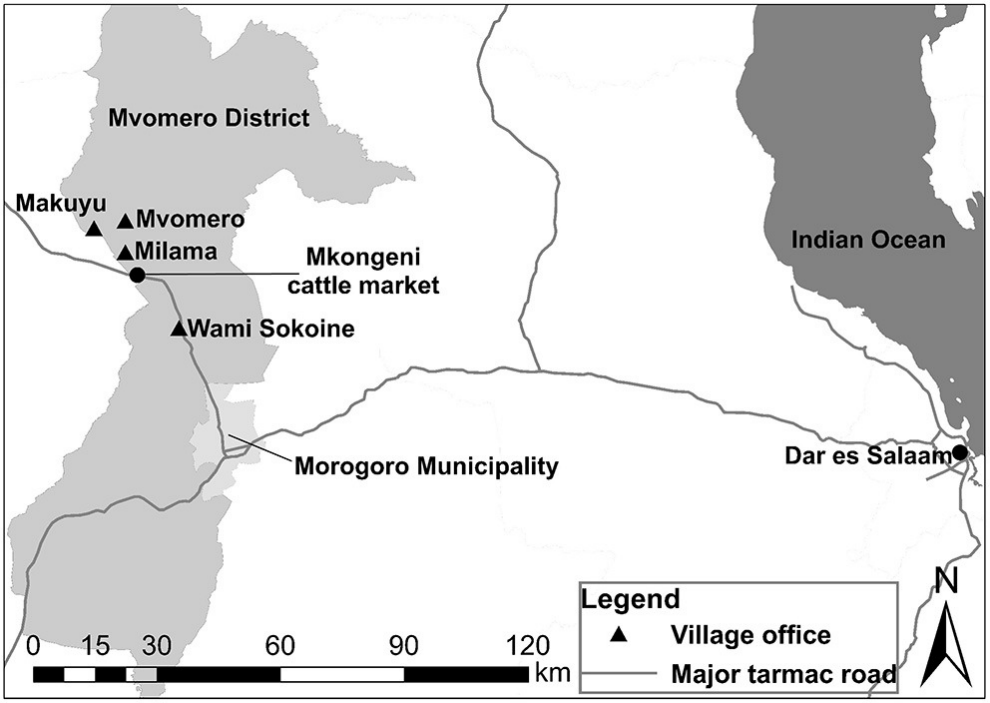
Map showing the locations of the villages surveyed in Mvomero District in Morogoro Region, Tanzania.

Four PRAs were conducted at the village offices of four agro-pastoral communities in the villages of Mvomero, Makuyu, Milama and Wami Sokoine in Mvomero District, Morogoro Region, Tanzania, between September and October 2017 (Figure 1). Quantitative brucellosis studies in cattle had been performed in the villages by the research team of this study (8, 9). The economy of the district is highly dependent upon agriculture. The main types of livestock raised in the villages are cattle, goats, sheep, pigs, donkeys and chickens. Most of the cattle farmers raise indigenous breeds using semi-extensive or extensive systems.

In addition to the research team, local administrative, veterinary, agriculture and medical officers were involved in administering the PRAs. The majority of participants in the PRAs were cattle farmers that were surveyed in our previous bovine brucellosis research conducted in 2016, as well as other cattle farmers (9). Women were encouraged to participate in the PRAs to ensure a gender-balanced view.

**Figure 2:**
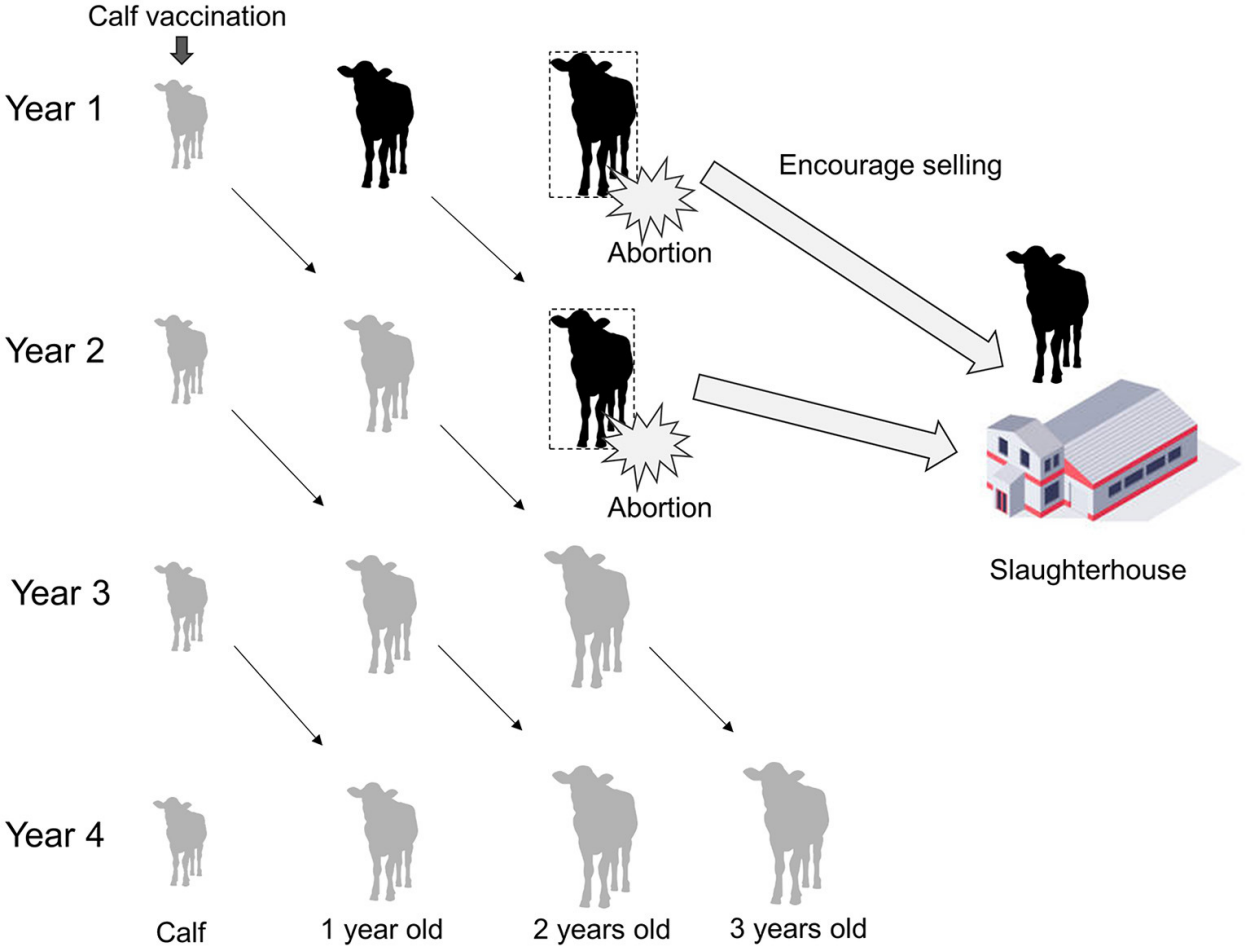
Schematic diagram showing a community-based brucellosis control plan involving selling cows with experience of abortion for slaughter and calf vaccination paid for by the farmers themselves. By following the plan, the proportion of immunized cows increases and that of brucellosis-infected cows decreases over time.

The PRAs were performed using Swahili, which is a national language in Tanzania, and English. Translation between the languages was done by Tanzanian researcher and local officers who were good at both languages. Voice recording of the PRAs was not conducted due to the communities' intentions. Thus, paper-based recording was used. Table 1 shows the checklist prepared for the PRAs; the checklist follows the manual on participatory epidemiology (19). The research team used the checklist as the basis of the PRAs, and always started with a self-introduction. After the self-introduction by research team and the participants, the characteristics of the disease in animals and humans were explained, and the results of previous studies on brucellosis prevalence in cattle, risk factor analyses for bovine brucellosis, and willingness-to-pay for the *Brucella* vaccine were explained (9). The participants were given the opportunity to ask questions in greater depth within the disease-associated topics. After the process, participants were asked to reflect on the set of questions raised by the research team. These questions focused on risky behaviors for human infection and possible brucellosis symptoms observed in their families. Then, the research team explained general state-led brucellosis control methods (mass vaccination, test and slaughter with compensation), the option of leaving the problem, and a potential community-based brucellosis control plan that included slaughtering cows with experience of abortion and vaccinating calves, with the cost of vaccination borne by the farmers themselves (Figure 2). After the procedure above, participants were encouraged to discuss about favorable disease control plan, as well as methods for reducing the risk of human infection. Farmers were able to ask any technical questions and to propose any other control options. At the end of the meetings, with the facilitation and the animation by the research team and local officers, participants were encouraged to express holistic approaches to community-based brucellosis control.

**Table 1:** Checklist used for the PRAs in this study.

**Items**	**Contents**
Self-introduction	Starting with investigators. Names and affiliations. Roles of officers.
Explanation about brucellosis	Causal agent, modes of infection, symptoms in humans and animals
Feedback of previous research findings on brucellosis	Prevalence and risk factors for bovine brucellosis, willingness to pay for vaccination
Customs associated with risky behaviors for brucellosis infection in humans	Drinking raw milk and cattle blood, facilitating parturition without protection against infection
Brucellosis symptoms within family	Undulant fever, headache, joint and back pain, fatigue
Explanation about brucellosis control methods, including community-based plan	Test and slaughter policy, limited diagnosis capacity in the area, mass vaccination, annual calf vaccination, and selling cows that have experienced abortion to slaughterhouse
Discussion about willingness to proceed with brucellosis control using a holistic approach	Facilitate discussions without guiding speakers

The activities basically followed the order shown in Table 1; however, when participants mentioned a topic that was further down the list, the flow of the discussion was changed to accommodate that topic. Nonetheless, care was taken to address all of the listed topics by the end of the meetings.

In addition, key informant interviews were performed with medical officers, veterinary officers and farmers at Mvomero District Medical Office, Mvomero District Veterinary Office, village offices in Mvomero and Morogoro Urban Veterinary Office, and livestock market, respectively. The interviews were based on free discussion on any issues associated with brucellosis and its control.

## Results

Table 2 shows a summary of the PRAs. The numbers of farmers who participated in the PRAs were 20, 15, 30 and 20 in Mvomero, Makuyu, Milama and Wami Sokoine villages, respectively. Women participated in all of the PRAs. A medical officer participated in the PRA in Makuyu village. The participants were comprised of several tribes; no Maasai were present at the meetings held in Mvomero and Makuyu villages, but Maasai comprised the majority of participants at Milama and Wami Sokoine villages. The information obtained through the PRAs is described below.

**Table 2:** General information about the farmers who participated in the PRA and a summary of the discussion about community-based disease control in each community.

**Village** **(No. of farmers who participated)**	**Tribal composition of the participants**	**Summary**
Mvomero (*n* = 20, male = 17, female = 3)	Several tribes and no Maasai	The community decided to proceed with the calf vaccination strategy, although the amount paid by each farmer for the vaccination differed among farmers, mainly depending on the number of cattle to be vaccinated; this was common to all of the other communities. The veterinary officers should play a major role in community-based disease control measures, especially in vaccination management; this was common to all of the other communities. Selling cows that had experienced abortion to be slaughtered is difficult due to a reduction in the selling price that occurs in response to negative information about the cattle; this was common to all of the other communities. Proposal to raise brucellosis-suspected cattle and healthy cattle separately to avoid the disease transmission.
Makuyu (*n* = 15, male = 13, female = 2)	Several tribes and no Maasai	Medical officers typically advised people to boil milk, but most of them did not. The officer commented the PRA held as part of this study may contribute to changing this behavior. Farmers discussed whether or not they could opt in of the community-based control measures by themselves, without the presence of research team.
Milama (*n* = 30, male = 28, female = 2)	Mainly Maasai	Proposal to change risky behaviors among children through education to prevent human infection was raised. The community basically agreed the community-based disease control. However, the chairman of the village also solicited opinions from other veterinary and livestock officers.
Wami Sokoine (*n* = 20, male = 17, female = 3)	Mainly Maasai	Participants expressed the opinion that all cattle farmers should participate in the community-based disease control. It was confirmed that the local veterinary officers and the farmers who participated in the PRA would share the plan with other members of the community.

### Risky Behaviors for Human Infection

Drinking raw cattle blood is customary among the Maasai, who consume cattle blood as an alternative to food and water especially during periods of nomadic herding. The Maasai believe that raw cattle blood provides a rich source of energy and that it removes harmful elements within the body.

Drinking raw milk is conducted by all tribes because they prefer the flavor and taste of raw milk compared to boiled milk. Insofar as gender and risky behavior are concerned, assisting with the birth of calves was performed by males, and females played a dominant role in milking especially among the Maasai. Farmers treated aborted materials with their bare hands, as plastic gloves were not available in the villages. The risk of human infection by risky behaviors was not recognized by the participants and knowledge of brucellosis was poor.

### Symptoms, Diagnosis and Treatment of Brucellosis in Humans

Suspected symptoms of brucellosis, such as undulant fever, headache, backache and muscular pain, were observed among the farmers and their family members. The local clinics did not have diagnostic equipment for brucellosis, and general symptomatic treatment was provided to patients who presented at clinics with brucellosis symptoms. Traditional remedies made from grasses or parts of trees were also used for treatment of febrile and pain related symptoms by households.

### Selling Cattle With Experience of Abortion

Most of the adult cattle that are traded at the Mkongeni market (Figure 1), which was the largest livestock market in the study area, were transported to large cities such as Dar es Salaam and Morogoro Municipality for slaughter. In addition, cattle trade among cattle farmers for raising purposes was also observed in and out of the market including personal trading. In the context of disease control, it is considered preferable to slaughter cows with experience of abortion, to eliminate the source of infection for other animals. However, it is difficult for the cattle farmers to control where the cows will be sent after they have been sold at the market. In addition, the selling price would be reduced if the dealer becomes aware of any negative information about the cows being sold.

### Calf Vaccinations Paid by Farmers

Since many of the farmers lacked knowledge of the *Brucella* vaccine, detailed information about the vaccine was provided to them. The cost of the vaccine was discussed frequently. Some farmers stated that vaccinating all of their calves may not be possible, especially if they have a large number of cattle; however, even in such cases, it may be possible to vaccinate selected cattle. The Makuyu community preferred to discuss the matter of the calf vaccination without the research team being present, and the discussion was undertaken in that way to protect their need for privacy. Finally, all of the communities reached the same conclusion and agreed that they would bear the cost of the calf vaccinations themselves, although it may not be possible for the farmers with large herd size to vaccinate all the calves to be vaccinated. Although the vaccination strategy was briefly accepted, the chairman of the Milama village was cautious and requested inputs from the other veterinary and livestock officers who were not participating in the PRA as a supportive information for decision making. Local veterinary officers were requested to be in charge of procurement of the vaccine.

### Holistic Approach Toward Brucellosis Control

In the PRAs, the rollout of the vaccination was also discussed and it was concluded that local veterinary officers were both suitably skilled and prepared to manage the process, and that they should also play a key role in the holistic community-based brucellosis control. There was a proposal to change the behaviors of children through education to prevent human infection, as changing traditional customs can be difficult for adults. Thus, from the perspective of health education, the involvement of schools and health facilities was regarded as important. In terms of how to disseminate a community-based disease control plan, the participants of the PRAs were encouraged to share the plan with their family members and other farmers.

### Key Informant Interviews

Table 3 shows the key information obtained from key informant interviews. The information which were not collected from the PRAs were listed.

**Table 3:** Key information obtained from key informant interviews.

**Interviewee**	**Information**
Mvomero District medical officer	Maasai rarely appear to medical facilities compared to other tribes, although they tend to conduct risky behaviors of *Brucella* infection.
Mvomero local medical officer	Many of febrile cases are diagnosed as malaria or typhoid fever. There must be misdiagnosis of brucellosis cases.
Morogoro Municipality veterinary officer	Cattle from agro-pastoral areas are slaughtered and consumed in urban areas. Therefore, brucellosis control in agro-pastoral areas is desirable even for urban areas.
Mvomero local veterinary officers	Veterinary officers guide farmers to boil milk before consumption, but farmers rarely do because of their preferences of taste and flavor of raw milk and unawareness of the risk of disease infection by raw milk consumption.
Farmers at market	It is commonly recognized among farmers that cattle traded at the markets may have problems such as diseases, infertility or poor growth so that they are on the market.

## Discussion

While this study was performed in agro-pastoral areas, our team has conducted brucellosis research since 2015 including urban areas in Morogoro Region. The research revealed that cattle raising system was different between the two areas: zero grazing, with small herd size and exotic dairy breeds in urban areas, and semi-extensive or extensive systems, with large herd size and indigenous breeds in agro-pastoral areas (8). In the comparative study, bovine brucellosis was quite limited in urban areas while prevalent in agro-pastoral areas, and higher chance of infection through grazing might be the reason for it (8). The Morogoro Municipality veterinary officers mentioned in a key informant interview that very low bovine brucellosis prevalence in urban areas was favorable, but since the disease was endemic in agro-pastoral areas and cattle from the areas were slaughtered and consumed in urban areas, the disease control in agro-pastoral areas is desirable even for urban areas (Table 3). Endorsed by the needs from urban areas as well, our team conducted quantitative research to investigate the possibility of community-based control using cattle vaccination. High willingness-to-pay had also been confirmed by the farmers in agro-pastoral areas (9).

This PE study was undertaken to assess whether community-based disease control is feasible under circumstances in which government-led disease control is challenging due to limited resources. The PRA revealed that drinking raw milk was common among all tribes, and drinking cattle blood was conducted only by the Maasai. This qualitative information was consistent with the results of a previous quantitative study, which reported that 66.7 and 48.4% of Maasai and other tribes consumed raw milk, and 63.3 and 0.0% consumed blood, respectively (9). Focusing on Maasai, previous study revealed that they had significantly higher brucellosis prevalence than other tribes (20). However, according to Mvomero District medical officer, they rarely appear to medical facilities (Table 3). Considering the Maasai traditional culture, in depth information about their sociological aspects should be investigated. Regarding the raw milk consumption, veterinary officers mentioned that although they guide farmers to boil milk before consumption, they rarely change the behavior because of their preferences of taste and flavor of raw milk and unawareness of the risk of raw milk consumption (Table 3). In terms of gender roles and raising cattle, males assisted with parturition of cows and females performed milking. Although the magnitude of the risks posed by these activities for human infection is unclear, since no significant gender difference in terms of disease prevalence was observed in human brucellosis in the study area (male: 29.9%, female: 38.2%, Odds ratio = 0.69, 95% CI: 0.33–1.45) (20), the main route of human infection was likely related to food consumption, as reported in previous studies (20, 21).

Farmers did not have a negative opinion regarding selling cows with experience of abortion. Although abortion in cattle can be caused by a variety of reasons, since abortion is strongly associated with bovine brucellosis in endemic areas, removing cows with experience of abortion is recommended (9, 22, 23). However, from a disease mitigation standpoint, selling potentially infected cattle has both positive and negative aspects. For example, while selling infected cattle may decrease the prevalence of brucellosis on farms, unless the infected cattle go to slaughterhouses, farmer-to-farmer cattle trades for raising purposes may contribute to the spread of brucellosis to other farms. In addition, selling potentially brucellosis-infected cattle to slaughterhouses may pose public health risks to slaughterhouse workers, meat inspectors, and consumers (7). In Tanzania, cattle that have been diagnosed with brucellosis cannot be sold for meat by law, but diagnosing all of the cattle that enter the food chain is not realistic. Thus, occupational risks for slaughterhouse workers may increase until the prevalence in animals decreases. However, as beef is typically cooked before for consumption, the public health risk posed by brucellosis from meat consumption is considered to be negligible. Further, in the absence of a national compensation scheme, selling potentially infected meat is a practical way for farmers to receive money for their animals and to mitigate brucellosis risk in cattle. Additional researches to evaluate the risks for occupation and consumption increased by proceeding selling potentially brucellosis-infected cattle and meat will determine the adequacy of the method. In addition, slaughtering high performance animals with history of abortion without brucellosis diagnosis may cause a serious issue particularly among commercial farms. Farmers mentioned their needs of diagnosis of their animals, and establishment of diagnostic service at farmers' cost should be considered.

Judging from the qualitative information obtained in the current study, the majority of cattle that are traded at the market are slaughtered rather than being sold and raised on another farm. In addition, according to the key informant interview to farmers, it is commonly recognized among farmers that cattle traded at the markets may have problems such as diseases, infertility or poor growth so that they are on the market (Table 3). Therefore, farmers are reluctant to buy cattle for raising purpose and this may be one of the reasons that most of the cattle traded at the market are slaughtered. In the PRAs, farmers argued that the decision of where to send the cattle that are sold at the livestock market lies with the buyers, and that disclosing that the cow had a history of abortion would decrease the selling cattle price. Although the disease-mitigation effect may outweigh the disease-spread effect, this selling policy of abortion-experienced cattle may increase inter-farm spread of the disease unless the authorities introduce some form of support.

Calf vaccination paid for by farmers themselves, which is at the center of the community-based brucellosis control plan, was accepted by all of the communities. This community-level agreement was in line with the quantitative results of a questionnaire survey which showed a high willingness among farmers to pay for calf vaccinations (9). For farmers who cannot afford to vaccinate all of their calves, we proposed that they only vaccinate new born calves as such a strategy would result in a gradual increase in vaccination coverage as the vaccination continues. This strategy would also spread the vaccination costs over time and be easier for farmers to accept. Although a rapid improvement is not expected using this calf-only vaccination strategy, slow but steady disease control, which is an important consideration in resource-limited situations, is expected over the long term. Moreover, a cost-benefit analysis of brucellosis vaccination would be helpful for decision making.

In the current study, we focused on the vaccination strategy for cattle among livestock. However, mixed livestock system especially raising sheep and/or goats along with cattle, which is very common in the study areas, was reported to be a risk factor for *Brucella* transmission between different animal species (8, 24–26). Thus, small ruminants should be included in the disease control strategy. Vaccination of sheep and goats has been successfully contributing to national brucellosis control and elimination strategies across Eastern Europe and Central Asia (27). In addition, it is reported in some countries that implementation of small ruminant vaccination reduced not only brucellosis in small ruminants and human, but also brucellosis in cattle as well, indicating that a larger proportion of bovine brucellosis is caused by *Brucella melitensis* infection than is commonly considered (27). A study conducted in Mvomero district showed the brucellosis prevalence in small ruminants was 1.4% (28), and another study reported detection of *B. abortus* from goats in Morogoro Region (29). Although the prevalence may be low, the degree to which *B. abortus* and *B. melitensis* epidemiology overlaps in mixed livestock system is unknown. Since brucellosis serological tests cannot distinguish the *Brucella* species, the isolation, identification and molecular characterization of *Brucella* spp. in the different livestock species and human are necessary to understand the transmission dynamics and to plan appropriate control measures (24). In addition, a study tried to understand cross-species *Brucerlla* transmission dynamics by integrating serological and genetic data, indicating the importance of the integration of multiple types of data (30). This kind of comprehensive study should be enhanced.

Interestingly, one of the communities discussed whether or not to participate in the community-based disease control scheme among themselves first, before informing the research team of their decision. It was considered that conducting discussions in this manner may encourage community members to speak freely and to exchange opinions honestly among themselves, leading to strong engagement and fostering a sense of responsibility for their decisions. Thus, regardless of their request, it may be better to provide participants with the opportunity to discuss such issues in meetings attended by community members only.

It was agreed in the PRAs that local veterinary officers would be in charge of vaccination management, and they would play an important role in the community-based brucellosis control. In the study areas, while working as public official, some veterinary officers have their own veterinary drug stores and sell medicines for animals, and provide veterinary medical treatment for livestock farmers. This indicates the incentives for veterinary officers in both public and private aspects in their social roles. Moreover, since farmers were not familiar with the vaccine and it was rarely used in the communities, the veterinary officer would not only be expected to manage the vaccine, but also to disseminate the correct knowledge about the vaccine and the vaccination program.

Human brucellosis is endemic to the study area where it has a prevalence of 33.3% (20). However, the diagnosis and specific treatment of human brucellosis are unfeasible in the studied communities due to the lack of materials and costs. A local medical officer mentioned that many of febrile cases were diagnosed as malaria or typhoid fever, indicating the misdiagnosis of brucellosis cases (Table 3). Consequently, prevention plays an important role in tackling human brucellosis in the area. In order to improve the knowledge, awareness and practice level of people for brucellosis, any disease control program should incorporate public health education to change high-risk behaviors and prevent human infection. The Tanzanian government has recently emphasized the importance of education and the number of children who attend school in the study area is increasing (9). The World Development Report identified school health programmes as among the most cost-effective of public health interventions (31). The primary reason is that the school setting itself offers a pre-existing and comprehensive system for health delivery: there are more teachers than nurses, more schools than clinics. In addition, health-related behaviors can be modified by interventions during the school-age years. Furthermore, the aims of health education directed at children are creating awareness about the existence of diseases, giving children practical skills in how to protect themselves and the community against diseases, and encouraging children's sense of responsibility for their own health and that of their families in the future (32). Thus, while it may be difficult to change traditional customs especially among the elderly, public health education for children in collaboration with education at school, public health and animal health authorities should be effective for changing risky behaviors and its sustainability.

This study was undertaken in 2017, which is 4 years ago at the time of writing, and there could be changes in behaviors among farmers and communities due to the influence by the PRAs. Fundamentally, community-based participatory research is a co-operative and co-learning process that facilitates the reciprocal transfer of knowledge and skills between communities and researchers (33, 34). Thus, future research should evaluate the effect of the PRAs in these communities and the findings should be shared among the stakeholders, and the co-learning process should be continued.

One of the limitations of this study was that, since the research team presented the disease control plan prior to discussions among the members of the communities themselves, the participatory disease control planning may be biased by the views that were initially presented by the team. However, information including conventional state-led brucellosis control was needed to initiate an informed discussion in the groups. Participants were also encouraged to ask any technical questions and propose any ideas of community-based brucellosis control. To overcome this limitation, additional research is considered necessary to collect more information and opinions about disease control from the communities themselves and stakeholders using a variety of different participatory approaches. Moreover, participation of local administrative, veterinary, agriculture, and medical officers might cause bias in the results. Generally, in the process of designing solutions with the community, it is appropriate to suggest components of the solution. In participatory epidemiology, it is recommended to firstly ask the community for ideas on ways to control the disease and understand how far they get. Then the facilitators can suggest options and guide the community to develop an effective and acceptable program. This process is referred to as community dialogue and is an interaction between the community and facilitators as equals to develop the intervention, which should be considered in additional researches (35, 36). This study provided the first information about the view of communities, but such participatory studies should be repeated to reach saturated consensus.

The findings of this study suggested that establishing a community-based brucellosis control plan in conjunction with public and animal health authorities is feasible, which confirms the correspondence between these qualitative results and previous quantitative studies. Further, if the holistic community-based brucellosis intervention is successfully implemented, these methods could potentially be applied to other countries where brucellosis is endemic. On the other hand, even if the disease control is implemented, cases of abortion in livestock and human febrile illness will still occur due to reasons other than brucellosis (37). In addition, the long period required to observe the clear effect, due to the slow increase of the vaccination coverage by calf vaccination, may distract communities from continuation of the program. Furthermore, considering the non-specific syndromes of human brucellosis, it might be difficult for the communities to recognize clear and tangible benefits of the intervention in a short period, which indicates the risk of loss in community's interest toward disease control during the implementation of it. Thus, understanding and clear communication of the multi-factorial causes of common disease syndromes are critical to prevent loss of trust by farmers. Moreover, the intervention should be supported by periodic communications about the perceptions of impact and expectations among the stakeholders, which makes possible to manage the risk of communities' distraction. The biggest effort should be paid to quantify the economic and public health benefit of brucellosis control, and to communicate it to farmers to gain the trust first (37).

## Data Availability Statement

The original contributions presented in the study are included in the article/supplementary material, further inquiries can be directed to the corresponding author.

## Ethics Statement

The studies involving human participants were reviewed and approved by Ethical Committee of the Graduate School of Dairy Science, Rakuno Gakuen University. Written informed consent for participation was not required for this study in accordance with the national legislation and the institutional requirements.

## Author Contributions

SA coordinated the research, performed field surveys, and wrote the majority of the manuscript. GM contributed to field surveys. KJ and RK coordinated the research. KM designed the research and contributed to field surveys and writing the manuscript. All authors contributed to the article and approved the submitted version.

## Funding

This work was supported by the Ministry of Education, Culture, Sports, Science and Technology of Japan as part of a research project titled, Development of rapid diagnostic kits for infectious pathogens in industry animals and establishment of effective control methods through global analysis of transmission routes, which was funded in part by a 2013 Support Grant for the Establishment of a Strategic Research Platform for Private Universities. SA thanks Japan International Cooperation Agency (JICA) and Japan Intellectual Support Network in Agricultural Sciences (JISNAS) for funding the preparatory phase of the study.

## Conflict of Interest

The authors declare that the research was conducted in the absence of any commercial or financial relationships that could be construed as a potential conflict of interest.

## Publisher's Note

All claims expressed in this article are solely those of the authors and do not necessarily represent those of their affiliated organizations, or those of the publisher, the editors and the reviewers. Any product that may be evaluated in this article, or claim that may be made by its manufacturer, is not guaranteed or endorsed by the publisher.
